# VEXAS syndrome presenting with severe cutaneous manifestations, inflammatory arthritis, and myelodysplastic syndrome treated with Ruxolitinib

**DOI:** 10.1093/omcr/omag121

**Published:** 2026-07-12

**Authors:** Jared Tesch, Nada Alrifai, Beth Honl, Siddharth Singhal

**Affiliations:** Department of Biological Sciences, North Dakota State University, 1340 Bolley Drive, Fargo, ND 58102, United States; Division of Rheumatic and Autoimmune Diseases, M Health Fairview Clinics and Surgery Center, Department of Medicine, University of Minnesota, 606 24th Avenue South, Suite 200, Minneapolis, MN 55454, United States; Dermatology Service, Veterans Affairs Health Care Systems, 2101 Elm St NE, Fargo, ND 58102, United States; Department of Medicine, Veterans Affairs Health Care Systems, 2101 Elm St NE, Fargo, ND 58102, United States; Department of Medicine, University of North Dakota School of Medicine and Health Sciences, 1301 N Columbia Rd, Grand Forks, ND 58203, United States

**Keywords:** VEXAS syndrome, autoinflammatory disorder, Ruxolitinib, inflammatory arthritis, myelodysplastic syndrome, cutaneous manifestations

## Abstract

VEXAS syndrome is a recently identified adult-onset autoinflammatory disorder caused by somatic mutations in the UBA1 gene, leading to systematic inflammation and hematological dysfunctions. We present the case of a 71-year-old man with a prolonged history of cutaneous lesions and inflammatory arthritis that is refractory. Despite the treatment with multiple immunosuppressive therapies, his symptoms persisted alongside macrocytic anemia and thrombocytopenia. Genetic testing confirmed a UBA1 mutation, which established the diagnosis of VEXAS syndrome. A subsequent bone marrow biopsy revealed myelodysplastic syndrome. This case highlights the importance of early recognition of VEXAS syndrome, particularly in patients with multisystemic involvement and treatment-refractory disease. Therapies such as JAK inhibitors may provide effective disease control in patients who are not candidates for hematopoietic stem cell transplantation.

## Introduction

VEXAS Syndrome (Vacuoles, E1 enzyme, X-linked, Autoinflammation, Somatic) is a recently described adult-onset autoinflammatory disorder first identified in 2020 by Beck et al. It is caused by acquired somatic mutations in the UBA1 (Ubiquitin-activating enzyme 1) gene arising within hematopoietic stem cells. These mutations impair ubiquitin activation, resulting in dysregulated overactivation of innate immune signaling and bone marrow dysfunction [1].

The resulting immune dysregulation drives chronic, relapsing systemic inflammation involving multiple organ systems, including the skin, joints, cartilage, lungs, and ocular structures. Hematologic involvement is common and may manifest with bone marrow failure, macrocytic anemia, thrombocytopenia, and increased risk of hematologic malignancies such as myelodysplastic syndrome (MDS).

This case highlights the critical role of multidisciplinary collaboration among dermatology, rheumatology, and hematology in diagnosing VEXAS syndrome. Our patient’s symptoms remained refractory to multiple disease-modifying antirheumatic drugs (DMARDs). His subsequent response to ruxolitinib sheds light on emerging treatment strategies, although allogeneic hematopoietic stem cell transplantation remains the only potentially curative treatment.

## Case report

**Figure 1:**
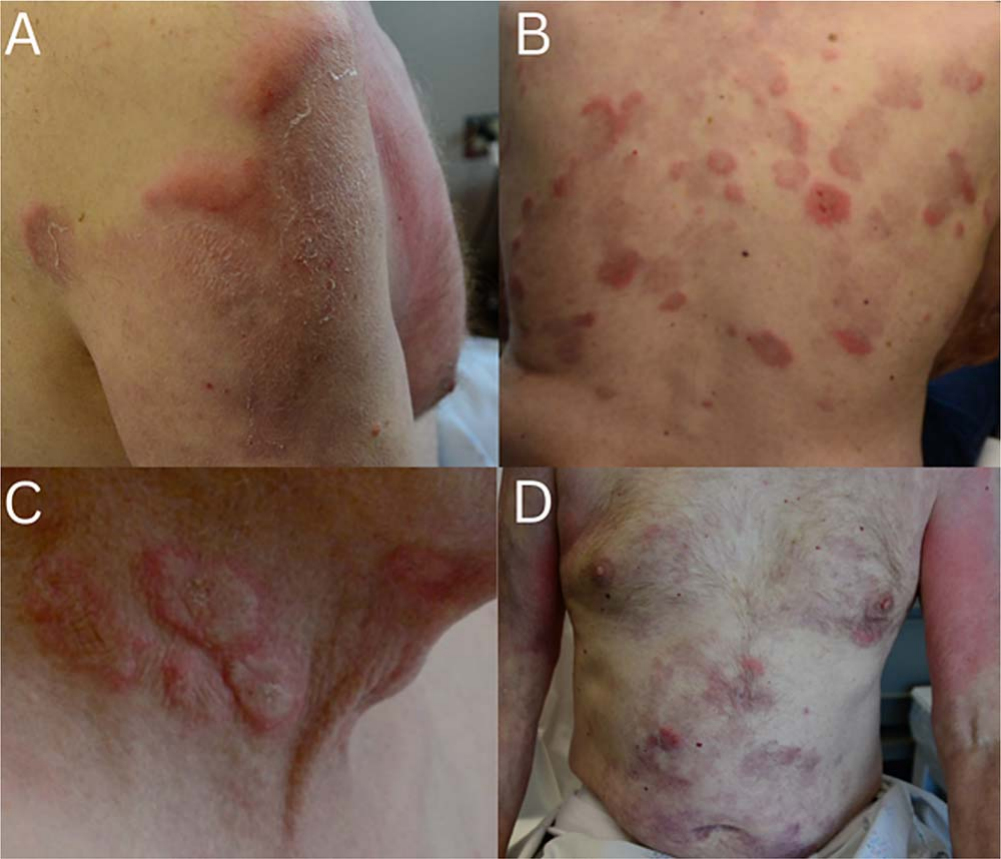
Widespread erythematous indurated annular plaques with violaceous borders and post-inflammatory changes in VEXAS syndrome.

**Figure 2:**
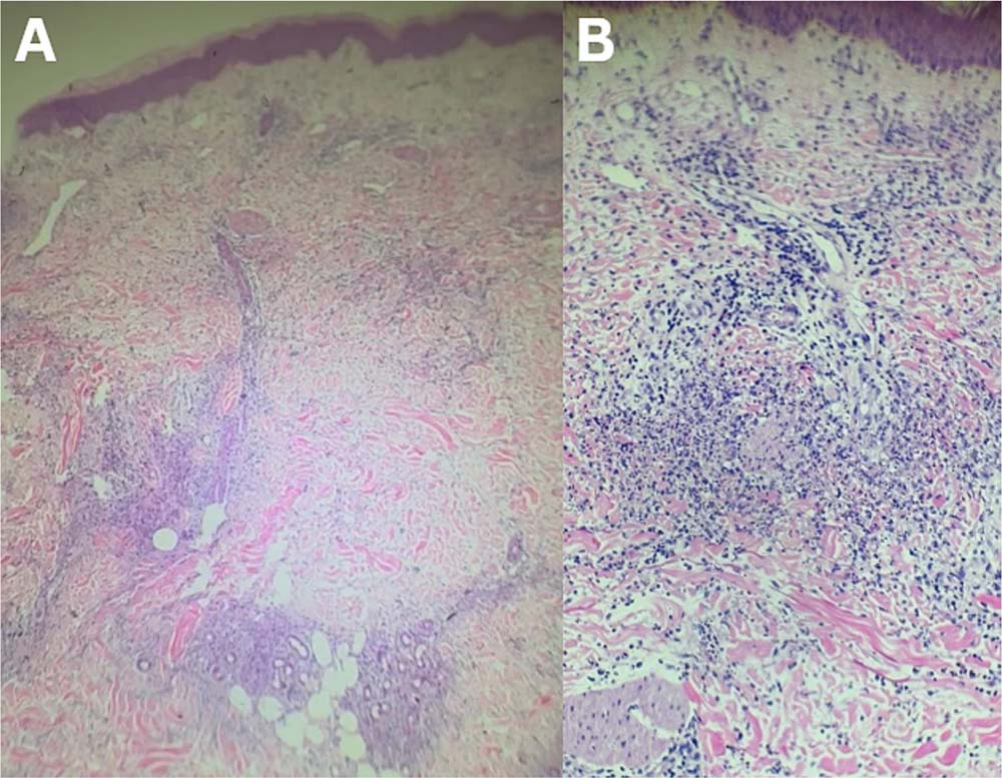
Histopathology of a punch biopsy from the left arm, stained with H&E. (A) Marked papillary dermal edema with a superficial and deep dermal inflammatory infiltrate (×40). (B) Higher power demonstrates dense neutrophilic infiltrates with prominent karyorrhectic debris (×100).

A 71-year-old Caucasian man with a past medical history of prostate cancer (on bicalutamide and finasteride), atrial fibrillation, and type 2 diabetes mellitus initially developed pruritic, ‘welt-like’ skin lesions involving the face, neck, and forearms in 2016. Approximately 18 months later, he developed oligoarticular arthritis. Over subsequent years, his cutaneous manifestations and inflammatory arthritis progressively worsened. In 2018, a skin biopsy indicated ‘deep form of erythema annulare centrifugum (gyrate erythema)’ and the patient was also diagnosed with ‘rheumatoid arthritis.’ Following the initial diagnoses, he was treated with numerous DMARDs for rheumatoid arthritis, including hydroxychloroquine, methotrexate, sulfasalazine, adalimumab, etanercept, azathioprine, tofacitinib, and rituximab. His inflammatory arthritis and cutaneous manifestations remained refractory. During treatment, he demonstrated partial responsiveness to systemic corticosteroids and required chronic prednisone therapy at a baseline dose of 15 mg daily, with intermittent higher doses of 20–30 mg daily during disease flares. He became steroid dependent. Multiple dermatologic evaluations described ‘widespread very large erythematous indurated annular plaques with violaceous borders or surrounding violaceous patches’, as well as ‘widespread post-inflammatory violaceous patches’ (Fig. 1). The patient also reported unintentional weight loss of approximately 40 pounds over several years. In 2023, a repeated skin biopsy demonstrated neutrophilic dermatosis with superficial and deep neutrophilic infiltrates and karyorrhectic debris (Fig. 2), consistent with Sweet’s syndrome or urticarial vasculitis, prompting a revised diagnosis. Clinically, the patient’s skin features included widespread post-inflammatory violaceous patches, which favored the diagnosis of urticarial vasculitis.

Extensive laboratory evaluation over several years revealed a negative infectious workup and unremarkable autoimmune serologies, including rheumatoid factor, cyclic citrullinated peptide (CCP) antibody, and antinuclear antibody (ANA). However, laboratory studies consistently demonstrated chronic macrocytic anemia, thrombocytopenia, and persistently elevated C-reactive protein (CRP). Vitamin B12 and folate levels were within normal limits. Despite treatment with numerous DMARDs, the patient’s symptoms remained refractory along with the persistent chronic macrocytic anemia and thrombocytopenia. Due to the complexity of his presentation, consultation with the Department of Rheumatology at the University of Minnesota was pursued, where the consulting rheumatologist suspected hematologic disorders such as MDS, along with systemic inflammation involving the skin and joints. VEXAS syndrome was then highly suspected, and UBA1 genetic testing via peripheral blood was ordered. A somatic UBA1 mutation (c. 121A > C; p. Met41Leu) was identified, confirming the diagnosis of VEXAS. Subsequent bone marrow biopsy revealed myelodysplastic syndrome with multilineage dysplasia (MDS-MLD) without increased blasts.

Following diagnosis, treatment with ruxolitinib, a Janus kinase (JAK) inhibitor, was initiated. Approximately one month after starting ruxolitinib, improvements in hemoglobin and platelet counts were observed, and further improvements were noted two months after the treatment (Table 1). During follow-up evaluations about two months after the treatment, there was no reported flare-up of joint pain, and skin inflammation had subsided.

**Table 1:** Hematologic laboratory values before and after initiation of ruxolitinib demonstrating improvement in hemoglobin and platelet counts following treatment.

Date	Hemoglobin (g/dl)	RBC (M/cmm)	MCV (fl)	Platelets (k/cmm)
02/2022	11.9	3.31	105.1	148
06/2023	10.5	3.18	99.1	155
12/2023	10.3	2.93	102.0	101
06/2024	10.9	3.31	98.2	129
09/2025	10.0	2.70	108.9	81
10/2025	9.1	2.50	108.8	90
12/2025 (1 month post-treatment)	11.3	3.02	109.9	174
01/2026 (2 months post-treatment)	11.8	3.38	105.3	215

## Discussion

VEXAS syndrome is frequently misdiagnosed because of its overlap with more common inflammatory, hematologic, and rheumatologic conditions. Our patient was treated for seronegative rheumatoid arthritis, disseminated granuloma annulare, and Sweet's syndrome, failing multiple immunosuppressive therapies before VEXAS was established. Prompt recognition is critical, as VEXAS syndrome may follow a fatal course, with reported mortality rates approaching 40% due to complications such as progressive anemia and respiratory failure among others [2].

Cutaneous involvement is highly prevalent, occurring in 88% of patients in one retrospective cohort [3]. Reported histopathologic patterns include neutrophilic urticarial dermatosis, leukocytoclastic or urticarial vasculitis, urticarial tissue reaction, neutrophilic dermatosis, neutrophilic panniculitis, and nonspecific chronic septal panniculitis [2]. Hematologic involvement is common, particularly macrocytic anemia [1, 2]. MDS develops in approximately 40% of patients [4]. In a systematic review, articular involvement was observed in 47.3% of patients, respiratory disease in 61.3%, and venous thromboembolism in 41.8% [5].

Diagnosis relies on the patient's history, phenotypic features, laboratory and bone marrow assessment, and is confirmed by identification of a pathogenic UBA1 mutation through genetic testing. Clinicians may consider the ‘SWIM Score’ as a screening tool to identify patients who warrant UBA1 genetic testing. This tool incorporates four equally weighted features: Skin involvement, Weight loss, Inflammation, and Macrocytic anemia [6]. In a study done by von Bornemann Fløe et al., a SWIM score ≥ 2 demonstrated 100% sensitivity in both derivation and validation cohorts [6]. Notably, this patient fulfilled all four SWIM criteria, strongly supporting early genetic testing for VEXAS syndrome. As such, the SWIM score may facilitate earlier diagnosis and promote timely initiation of appropriate management.

Although allogeneic hematopoietic stem cell transplantation (HSCT) remains the only curative treatment, emerging data suggest that targeted therapies such as JAK inhibitors may provide meaningful disease control [7]. Moreover, HSCT is often not feasible, as most patients are older adults with comorbidities that increase infectious and transplant-related mortality risk [8]. Other proposed treatments include DNA hypomethylating agents such as azacitidine and interleukin-6 inhibitors such as tocilizumab [9]. In this patient, ruxolitinib led to improvements in cell counts within one month of initiation at a dose of 5 mg twice daily. Although macrocytosis persisted, dose optimization is ongoing with further hematologic and inflammatory improvement observed during post treatment follow-ups.

The prolonged disease course reflected in our case may reflect the specific UBA1 variant identified, p.Met41Leu. This is the rarest of the three pathogenic codon 41 substitutions and has been associated with a milder presentation as compared to p.Met41Val and p.Met41Thr. A large-scale multicentre analysis of 116 French VEXAS patients demonstrated notably worse survival among those with the p.Met41Val mutation, while p.Met41Leu and p.Met41Thr showed comparatively better outcomes [4]. These associations may explain the near-decade of symptomatic disease prior to diagnosis and show that the reported mortality rates reflect the broader VEXAS syndrome population rather than certain variant subtypes.
